# Heterozygous frameshift mutation in keratin 5 in a family with Galli–Galli disease

**DOI:** 10.1111/bjd.12813

**Published:** 2014-06-19

**Authors:** AK Reisenauer, SV Wordingham, J York, EWJ Kokkonen, WHI Mclean, NJ Wilson, FJD Smith

**Affiliations:** 1 Kaiser Permanente1279 S. Kihei Rd, Kihei, HI, U.S.A; 2 Centre for Dermatology and Genetic Medicine, Colleges of Life Sciences and Medicine, Dentistry and Nursing, University of DundeeDundee, DD1 5EH, U.K; 3 Southern Illinois University School of MedicineSpringfield, IL, U.S.A

## Abstract

**Background:**

Reticulate pigmentary disorders include the rare autosomal dominant Galli–Galli disease (GGD) and Dowling–Degos disease (DDD). Clinical diagnosis between some of the subtypes can be difficult due to a degree of overlap between clinical features, therefore analysis at the molecular level may be necessary to confirm the diagnosis.

**Objectives:**

To identify the underlying genetic defect in a 48-year-old Asian-American woman with a clinical diagnosis of GGD.

**Methods:**

Histological analysis was performed on a skin biopsy using haematoxylin–eosin staining. *KRT5* (the gene encoding keratin 5) was amplified from genomic DNA and directly sequenced.

**Results:**

The patient had a history of pruritus and hyperpigmented erythematous macules and thin papules along the flexor surfaces of her arms, her upper back and neck, axillae and inframammary areas. Hypopigmented macules were seen among the hyperpigmentation. A heterozygous 1-bp insertion mutation in *KRT5* (c.38dupG; p.Ser14GlnfsTer3) was identified in the proband. This mutation occurs within the head domain of the keratin 5 protein leading to a frameshift and premature stop codon.

**Conclusions:**

From the histological findings and mutation analysis the individual was identified as having GGD due to haploinsufficiency of keratin 5.

**What's already known about this topic?**

- Mutations in keratin 5 have been identified as an underlying cause of Galli–Galli disease (GGD).

**What does this study add?**

- Identification of a previously unreported frameshift mutation in keratin 5 resulting in a premature stop codon provides further evidence that GGD is caused by haploinsufficiency of keratin 5.

Galli–Galli disease (GGD) is a rare autosomal dominant condition in the class of reticulate pigmentation disorders. It is an allelic variant of the clinically similar disorder, Dowling–Degos (DDD, OMIM 179850), but with the reported distinguishing histological feature of acantholysis. However, the number of cases described is small and acantholysis has also been observed in cases with DDD.^1^ Patients classically present with pigmented lesions involving large body folds and flexural areas. In rare cases, the face can be involved and comedonal-like papules and pitted scarring can be seen. GGD was first described in 1982 when two brothers were reported to have a histologically distinct disease, which included acantholysis.^2^ Since then a number of cases of both DDD and GGD have been reported in the literature associated with mutations in keratin 5 (encoded by the *KRT5* gene).^1^,^3^–^8^ Herein, we report the case of an Asian-American woman with a clinical diagnosis of GGD and a previously unreported mutation within *KRT5*.

## Materials and methods

A 4-mm punch biopsy was taken from the right axilla for histological analysis by haematoxylin–eosin (H&E) staining. Genomic DNA was obtained with informed consent and appropriate ethical approval that complies with the Declaration of Helsinki principles. All exons and intron/exon boundaries of *KRT5* were amplified using primers specific to *KRT5* and polymerase chain reaction products were directly sequenced (Data S1; see Supporting Information).

## Results

### Clinical features

**Fig. 1:**
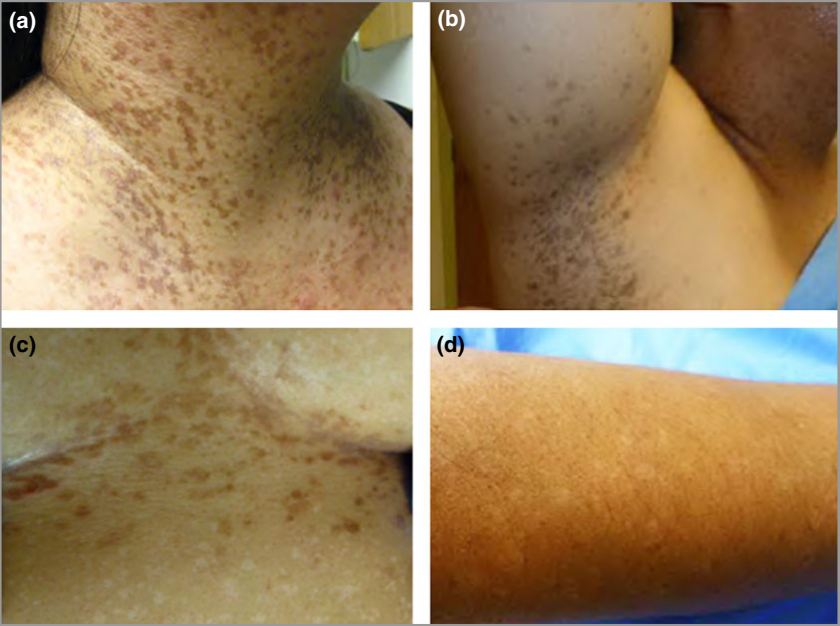
Clinical features of Galli–Galli disease. Hyperpigmented slightly scaly papules coalescing in a reticulate fashion over flexural areas, including (a) the anterior neck and (b) axillae; (c) reddish-brown thin papules (5–9 mm) in the inframammary area with multiple 4–7-mm hypopigmented macules scattered on the abdomen; (d) hypopigmented Galli–Galli disease macules on the forearm.

A 48-year-old Asian-American woman presented with a 20-year history of pruritus and an ever-present rash. Initially the rash was present on the flexural surface of her arms, and gradually spread to involve her neck, axillae and breast folds. The pruritus characteristics were made worse on extreme heat and perspiration. There were no relieving factors reported and no history of pain or photosensitivity. Physical examination revealed hyperpigmented and erythematous macules and thin scaly papules coalescing into plaques along the flexor surfaces of her arms, her upper back and neck, axillae and inframammary areas (Fig. 1). Amidst the hyperpigmentation were scattered hypopigmented macules. She also had a discrete area of hypopigmentation on her abdomen. Comedonal-like papules were present on her chin and perioral area while there were focal areas of pitted scarring on her cheeks. She was initially prescribed hydrocortisone cream 2·5% twice daily and tretinoin cream 0·025% once nightly. The tretinoin cream was subsequently stopped because of skin irritation. Subsequently she has used hydrocortisone cream twice weekly and daily emollients as needed.

### Histology and mutation analysis

**Fig. 2:**
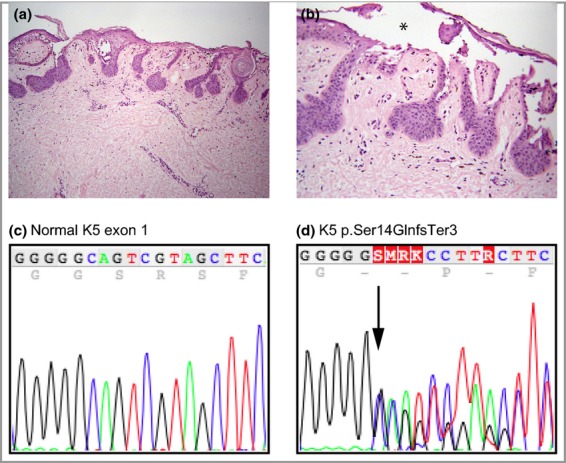
(a,b) Histopathological features of Galli–Galli disease. (a) A biopsy from the right axilla reveals sparse superficial to mid-dermal perivascular lymphocytic infiltrate with papillary dermal melanophages. Foci of intraepidermal acantholysis can mimic focal acantholytic dyskeratosis. Haematoxylin–eosin (H&E) staining; original magnification: ×10. (b) Extensive suprabasilar acantholysis leads to an intraepidermal split, indicated by the asterisk. H&E staining; original magnification: ×20. (c,d) Mutation analysis. (c) Normal *KRT5* sequence in exon 1, showing nucleotides 34–51. (d) The region equivalent to that in (a) from the proband showing heterozygous mutation c38dupG (arrow) leading to a premature stop codon, Ser14GlnfsTer3. K5, keratin 5.

Histological analysis of a biopsy showed lentiginous epidermal hyperplasia with elongated rete ridges and suprapapillary plate thinning, focal orthokeratosis, small milia-like cysts and keratin plugs, and suprabasilar acantholysis (Fig. 2a,b).

A heterozygous 1-bp insertion mutation in exon 1 of *KRT5* (c.38dupG; Ser14GlnfsTer3) was identified in the proband leading to a frameshift and premature stop codon two amino acids downstream (Fig. 2c,d). The mutation occurred within the head domain of the keratin 5 polypeptide and is predicted to lead to haploinsufficiency of keratin 5. The mutation was excluded from two unaffected family members (her 28-year-old daughter and 16-year-old son) and 90 control DNA samples from unrelated white subjects using fluorescent DNA fragment analysis. This variant is not present on the Exome Variant Server [NHLBI GO Exome Sequencing Project (ESP), Seattle, WA, U.S.A.; http://evs.gs.washington.edu/EVS/] nor on the dbSNP database.

## Discussion

Mutations in the *KRT5* gene have been reported since the early 1990s as the cause of epidermolysis bullosa simplex (EBS).^9^ Keratin 5, a type II keratin protein, partners with the type I keratin 14 and this keratin pair is predominantly expressed within the basal cells of the epidermis where blistering in EBS occurs. There is some genotype–phenotype correlation between the dominant-negative mutations in *KRT5* that cause EBS; the severe Dowling–Meara form is normally due to mutations in the highly conserved helix boundary domains at either end of the rod domain, while mutations throughout the rod domain tend to result in milder forms of EBS. The subtype of EBS associated with mottled pigmentation (EBS-MP) is most often due to a specific mutation, p.Pro25Leu, in the head domain of keratin 5.^9^–^11^ The *KRT5* mutations reported in GGD and DDD are also within the head domain but are nonsense or frameshift mutations resulting in premature termination codons; these lead to haploinsufficiency of keratin 5 rather than a dominant-negative defect.^1^

The hyperpigmentation observed in disorders such as EBS-MP, GGD or DDD that is associated with mutations in *KRT5*, has led to a growing body of evidence that keratin 5 may also play a role in the function or transportation of melanosomes. How melanosomes are transferred from melanocytes to keratinocytes is still unclear although a number of mechanisms have been proposed.^12^ How keratin 5 might be involved in this process remains unknown.

Although many cases of the main reticulate pigmentary disorders can be characterized by their clinical and histopathological features, there is a certain degree of overlap between the subtypes of reticulate pigmentary disorders, for example between DDD and the acantholytic variant GGD, making diagnosis difficult.^13^ Analysis at the molecular level is useful to confirm the subtype and identify allelic variants.^13^ GGD is due to mutations in *KRT5*; DDD is caused by mutations in *KRT5* or *KRT14* (http://www.interfil.org),^9^ and most recently it has been shown some cases are due to mutations in *POFUT1* (which encodes protein O-fucosyltransferase 1).^14^ Reticulate acropigmentation of Dohi is associated with mutations in *ADAR1* (adenosine deaminase, RNA-specific 1) on chromosome 1, and mutations in *ADAM10*, encoding a zinc metalloprotease, have recently been identified in several kindreds with reticulate acropigmentation of Kitamura;^15^ at present there are no genes/gene loci associated with Haber syndrome.^13^ As already reported, with the advent of whole-exome sequencing at least two new genes associated with reticulate pigmentary disorders have been identified, *POFUT1* and *ADAM10*, and further genes may still be identified in cases with reticulate pigmentation with no mutation to date in a known gene.

This work expands the spectrum of known mutations in *KRT5* and strengthens the body of evidence that GGD is caused by haploinsufficiency of keratin 5.

## Supporting Information

**Data S1.** Mutation detection.
