# Supplementary material for: Heterozygous frameshift mutation in keratin 5 in a family with Galli–Galli disease
Source: Br J Dermatol. 2014 Jun 19;170(6):1362–5. doi: 10.1111/bjd.12813 (PMC4150463; doi:10.1111/bjd.12813)
Supplement: Supplementary file 1 — Data S1. Mutation detection. [file bjd0170-1362-SD1.docx]

**Supporting Information**

**Data S1.** Mutation detection.

ATT TAT TTC AGA CCC ACA GT 3’ were used in Qiagen Coral Load PCR buffer containing 1.5mM MgCl_2_ and 1U HotStarTaq *Plus* DNA Polymerase (Qiagen, Crawley, UK). The following PCR conditions were used (95°C 5 min) x1; (94°C 1 min, 60°C 1 min, 72°C 1 min) x 35; and (72°C 10 min) x 1. PCR products were purified using ExoSAP (Exonuclease 1 and Shrimp Antartic Phosphatase, New England Biolabs) and sequenced on an ABI 3700 Automated DNA sequencer (Foster City, CA) according to the manufacturer’s instructions.

The mutation was excluded from unaffected family members and 89 unrelated Caucasian control DNA samples (178 chromosomes) by fluorescent DNA Genomic DNA was obtained with informed consent and appropriate ethical approval that complies with the Declaration of Helsinki Principles. Genomic DNA was extracted from peripheral blood lymphocytes using the Gentra PurGene method. All exons and intron/exon boundaries of *KRT5* were amplified using primers specific to *KRT5*. Primer sequences are available on request. For exon 1 forward primer 5’ CTC TCC AGC ACC TCC CAA CC 3’ and reverse primer 5’ GGC fragment analysis. Forward primer 5’ 6-FAM - GGG GGC ATC ACC GTT CCT 3’ and reverse primer 5’ GTT TCT TCT GCC ATA GCC ACC CAC TCC 3’ were used in Qiagen Coral Load PCR buffer containing 1.5mM MgCl_2_ and 1U HotStarTaq *Plus* DNA Polymerase (Qiagen, Crawley, UK). The forward primer was labelled with 6-FAM, and the ‘GTTTCTT’ sequence was added to the 5’ end of the reverse primer to improve adenylation of 3’ end of PCR product. The following PCR conditions were used (95°C 5 min) x1; (94°C 1 min, 60°C 1 min, 72°C 1 min) x 35; and (72°C 10 min) x 1. PCR products were diluted and analysed on an ABI 3100 Automated DNA sequencer.
